# Supplementary material for: The Barcelona Predictive Model of Clinically Significant Prostate Cancer
Source: Cancers (Basel). 2022 Mar 21;14(6):1589. doi: 10.3390/cancers14061589 (PMC8946272; doi:10.3390/cancers14061589)
Supplement: Supplementary file 1 [file cancers-14-01589-s001.zip › cancers-1632477-supplementary.pdf]

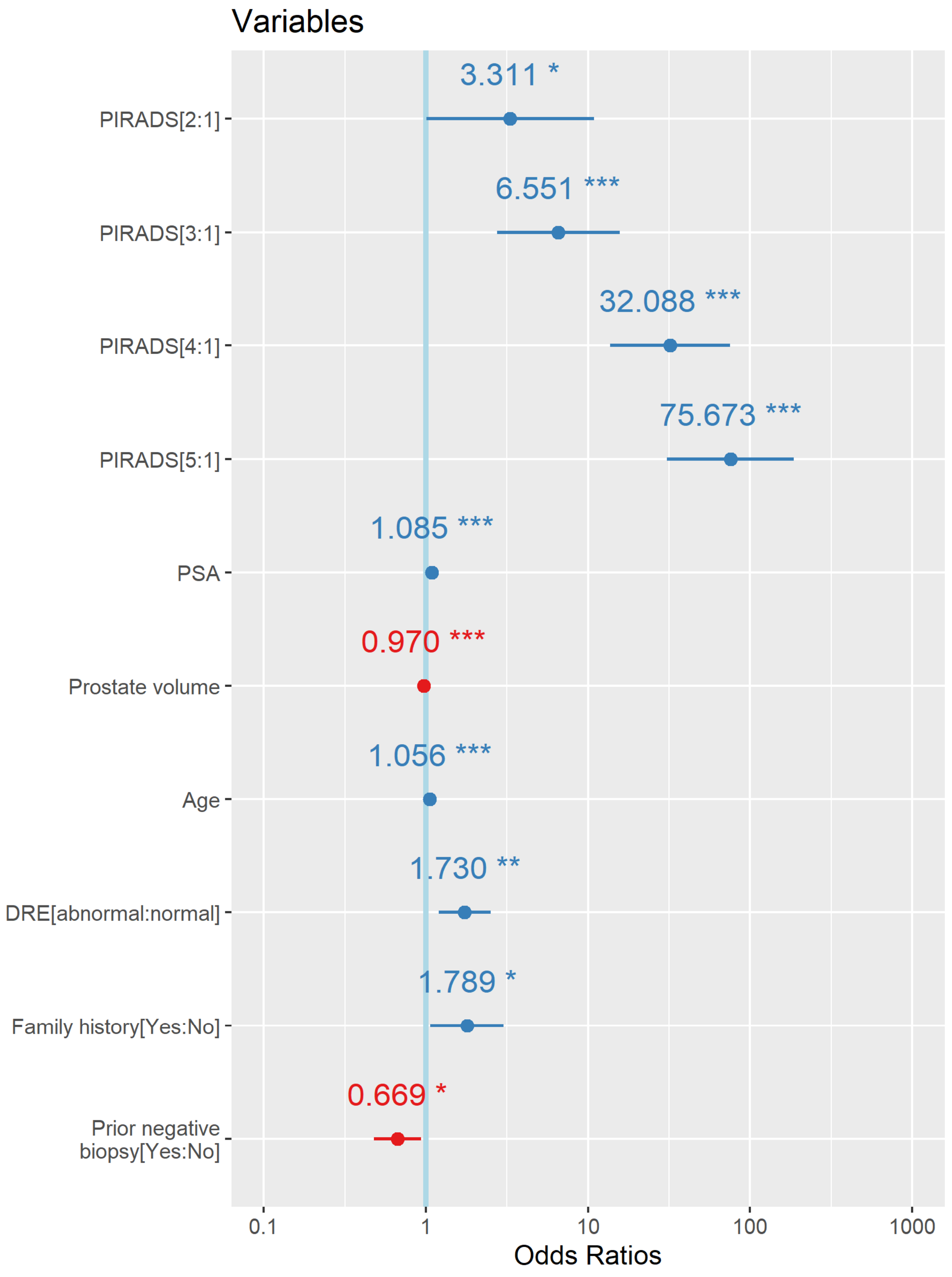

Figure S1. Forest plot of the odds ratios of independent predictors of csPCa included in the development cohort. PI-RADS = Prostate Imaging-Reporting and Data System; PSA = prostate-specific antigen; DRE = digital rectal examination; $*p = 0.05$, $**p = 0.01$, $***p < 0.001$

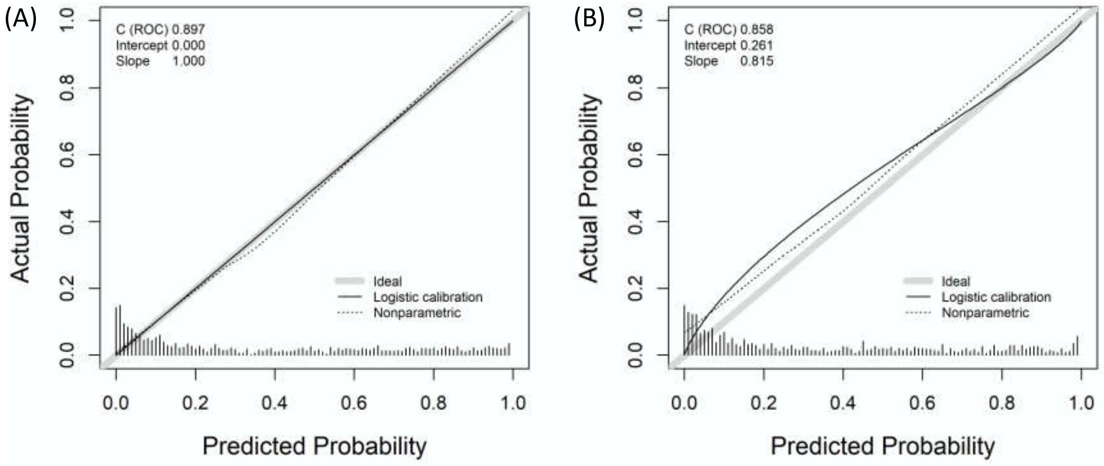

Figure S2. Calibration curve of MRI-PM in development cohort (**A**), and external validation cohort (**B**).

Table S1. Estimation, in a 1000 cases development (A) and external validation (B) cohorts of absolute missed csPCa and avoided biopsies for different thresholds and PI-RADS categories.

**Series**	DEVELOPMENT COHORT (A)										VALIDATION COHORT (B)									
**Group**	PIRADS 1-2		PIRADS 3		PIRADS 4		PIRADS 5		Global cohort		PIRADS 1-2		PIRADS 3		PIRADS 4		PIRADS 5		Global cohort	
Cases	*csPC* *9*	*total* *212*	*csPC* *46*	*total* *299*	*csPC* *159*	*total* *303*	*csPC* *155*	*total* *186*	*csPC* *369*	*total* *1000*	*csPC* *44*	*total* *248*	*csPC* *43*	*total* *212*	*csPC* *215*	*total* *413*	*csPC* *106*	*total* *126*	*csPC* *408*	*total* *999*
**Cut-off point**	Missed csPC (n)	Avoided Bx (n)	Missed csPC (n)	Avoided Bx (n)	Missed csPC (n)	Avoided Bx (n)	Missed csPC (n)	Avoided Bx (n)	Missed csPC (n)	Avoided Bx (n)	Missed csPC (n)	Avoided Bx (n)	Missed csPC (n)	Avoided Bx (n)	Missed csPC (n)	Avoided Bx (n)	Missed csPC (n)	Avoided Bx (n)	Missed csPC (n)	Avoided Bx (n)
**0**	0	0	0	0	0	0	0	0	0	0	0	0	0	0	0	0	0	0	0	0
**1**	1	47	0	9	0	0	0	0	1	56	3	45	0	6	0	0	1	1	4	52
**2**	3	99	0	15	0	1	0	0	3	115	4	86	0	11	0	0	1	2	5	99
**3**	3	131	0	20	0	1	0	0	3	152	10	119	1	18	0	2	1	2	12	141
**4**	5	151	1	33	0	1	0	0	6	185	14	151	2	26	1	5	1	2	18	184
**5**	5	164	1	51	0	1	0	0	6	216	15	162	3	37	1	7	1	2	20	208
**6**	5	174	1	64	0	3	0	0	6	241	18	181	3	44	1	7	1	2	23	234
**7**	5	182	5	79	0	3	0	0	10	264	20	191	4	57	1	8	1	2	26	258
**8**	5	187	7	92	0	3	0	0	12	282	23	202	4	71	1	13	1	2	29	288
**9**	5	192	7	106	0	4	0	0	12	302	26	207	4	78	1	14	1	2	32	301
**10**	5	194	9	118	1	7	0	0	15	319	30	216	4	89	1	16	1	2	36	323
**11**	5	196	11	136	1	8	0	1	17	341	32	224	5	101	1	18	1	3	39	346
**12**	5	199	11	153	1	11	0	1	17	364	33	225	6	111	2	20	1	3	42	359
**13**	6	201	11	167	1	11	0	1	18	380	34	226	6	123	2	25	1	3	43	377
**14**	6	202	13	177	1	11	0	1	20	391	35	228	6	131	3	26	1	3	45	388
**15**	6	203	13	185	1	12	0	1	20	401	36	232	6	134	4	30	1	3	47	399
**16**	7	205	15	196	1	13	1	1	24	415	36	234	8	142	4	37	1	3	49	416
**17**	7	206	16	202	2	15	1	1	26	424	38	236	10	146	4	42	1	3	53	427
**18**	7	206	16	208	3	17	1	2	27	433	39	239	13	151	4	47	1	3	57	440
**19**	7	207	16	216	3	22	1	2	27	447	40	240	14	155	4	52	1	3	59	450
**20**	7	207	20	223	3	25	1	2	31	457	40	240	14	159	4	55	1	3	59	457
**21**	7	207	21	229	4	26	1	3	33	465	40	240	16	164	5	61	1	3	62	468
**22**	7	208	24	236	4	29	1	3	36	476	40	240	16	167	6	64	1	3	63	474
**23**	7	209	27	241	5	31	1	3	40	484	40	241	18	173	7	70	1	3	66	487
**24**	7	209	27	242	5	34	1	3	40	488	40	241	19	177	8	74	1	3	68	495
**25**	7	209	28	248	5	36	1	3	41	496	40	242	19	177	8	77	1	3	68	499
**26**	7	209	31	258	5	39	1	3	44	509	41	243	19	177	10	85	1	3	71	508
**27**	7	209	31	261	6	44	1	3	45	517	41	244	19	180	10	87	1	3	71	514
**28**	8	210	31	263	6	46	1	3	46	522	41	244	20	183	13	94	1	3	75	524
**29**	8	210	32	266	7	50	1	3	48	529	41	244	21	185	15	98	1	3	78	530
**30**	8	210	33	268	9	57	1	3	51	538	41	244	23	188	17	100	1	3	82	535
**31**	8	211	35	271	9	59	1	3	53	544	41	244	29	193	17	104	1	3	88	544
**32**	8	211	36	272	9	61	1	3	54	547	42	245	31	196	19	109	1	3	93	553
**33**	8	211	36	273	11	63	1	3	56	550	42	245	31	199	19	111	1	3	93	558
**34**	8	211	37	277	12	65	1	3	58	556	42	245	31	199	21	115	1	4	95	563
**35**	8	211	37	277	13	66	1	3	59	557	42	245	32	200	21	118	1	4	96	567
**36**	8	211	38	279	13	67	1	3	60	560	42	246	34	202	23	123	1	4	100	575
**37**	8	211	40	281	13	71	1	3	62	566	42	246	34	202	24	126	1	4	101	578
**38**	8	211	41	283	16	74	1	3	66	571	42	246	34	202	24	130	1	4	101	582
**39**	8	211	43	285	17	79	1	3	69	578	42	246	34	202	25	135	1	4	102	587
**40**	8	211	43	285	19	86	1	3	71	585	42	246	36	204	26	137	1	5	105	592
**41**	8	211	43	285	20	90	1	3	72	589	42	246	38	206	30	145	1	5	111	602
**42**	8	211	43	285	22	93	1	4	74	593	42	246	38	207	33	152	1	5	114	610
**43**	8	211	43	286	22	98	1	4	74	599	43	247	38	207	34	158	1	5	116	617
**44**	8	211	43	287	22	101	1	4	74	603	43	247	38	207	34	160	1	5	116	619
**45**	8	211	43	288	23	104	1	4	75	607	43	247	38	207	36	164	1	5	118	623
**46**	8	211	43	289	26	109	1	4	78	613	43	247	39	208	44	178	1	5	127	638
**47**	8	211	43	289	28	114	3	6	82	620	43	247	39	208	45	182	1	6	128	643
**48**	8	211	43	289	32	123	3	6	86	629	43	247	39	208	47	187	2	7	131	649
**49**	8	211	43	289	34	129	3	6	88	635	44	248	39	208	50	196	2	7	135	659

**50**	8	211	43	289	36	137	4	7	91	644	44	248	39	208	52	202	2	7	137	665
**51**	8	211	43	289	39	142	4	7	94	649	44	248	39	208	59	209	2	7	144	672
**52**	8	211	43	290	43	147	5	7	99	655	44	248	39	208	63	216	2	7	148	679
**53**	8	211	44	291	44	150	5	7	101	659	44	248	39	208	69	223	2	8	154	687
**54**	8	211	44	291	44	151	5	7	101	660	44	248	39	208	71	226	3	10	157	692
**55**	8	211	44	293	48	159	5	7	105	670	44	248	39	208	72	232	4	12	159	700
**56**	8	211	44	293	49	164	5	8	106	676	44	248	39	208	74	237	4	12	161	705
**57**	8	211	44	293	53	170	5	9	110	683	44	248	39	208	77	243	5	13	165	712
**58**	8	211	44	294	55	175	5	9	112	689	44	248	39	208	79	247	5	13	167	716
**59**	8	211	44	294	59	180	5	12	116	697	44	248	39	208	84	255	6	14	173	725
**60**	8	211	44	295	63	186	5	12	120	704	44	248	39	208	87	262	6	14	176	732
**61**	8	211	45	296	66	190	7	15	126	712	44	248	40	209	89	266	7	16	180	739
**62**	8	211	45	296	70	194	7	15	130	716	44	248	41	210	92	272	7	16	184	746
**63**	8	211	45	296	73	199	9	20	135	726	44	248	41	210	93	275	10	19	188	752
**64**	8	211	46	297	77	204	9	21	140	733	44	248	41	210	97	280	10	19	192	757
**65**	8	211	46	297	79	209	10	22	143	739	44	248	41	210	99	282	11	21	195	761
**66**	8	211	46	297	83	215	10	24	147	747	44	248	41	210	107	294	11	21	203	773
**67**	8	211	46	297	91	224	11	26	156	758	44	248	41	210	107	295	12	22	204	775
**68**	8	211	46	297	94	229	11	26	159	763	44	248	41	210	113	301	13	23	211	782
**69**	8	211	46	297	97	232	12	28	163	768	44	248	41	210	115	303	14	25	214	786
**70**	8	211	46	297	100	236	14	30	168	774	44	248	41	210	116	307	14	25	215	790
**71**	8	211	46	297	104	240	14	31	172	779	44	248	41	210	121	312	14	25	220	795
**72**	8	211	46	297	106	244	15	32	175	784	44	248	41	210	126	317	15	27	226	802
**73**	8	211	46	298	108	248	15	32	177	789	44	248	41	210	129	320	16	29	230	807
**74**	8	211	46	298	114	253	18	35	186	797	44	248	41	210	134	326	17	30	236	814
**75**	8	211	46	298	120	260	20	38	194	807	44	248	41	210	136	329	17	30	238	817
**76**	8	211	46	298	122	264	23	40	199	813	44	248	41	210	140	334	20	34	245	826
**77**	8	211	46	298	124	266	24	42	202	817	44	248	41	210	142	336	20	34	247	828
**78**	8	211	46	298	128	271	28	46	210	826	44	248	41	210	144	338	23	37	252	833
**79**	8	211	46	298	132	275	30	49	216	833	44	248	42	211	150	346	25	39	261	844
**80**	8	211	46	298	135	279	34	53	223	841	44	248	42	211	153	349	26	40	265	848
**81**	8	211	46	298	138	282	40	59	232	850	44	248	42	211	155	351	27	42	268	852
**82**	8	211	46	298	140	284	43	62	237	855	44	248	42	211	161	356	27	42	274	857
**83**	8	211	46	298	141	285	50	69	245	863	44	248	42	211	166	362	32	48	284	869
**84**	8	211	46	298	143	287	55	77	252	873	44	248	42	211	174	370	33	50	293	879
**85**	8	211	46	298	145	289	61	83	260	881	44	248	42	211	177	372	36	53	299	884
**86**	8	211	46	298	146	290	66	89	266	888	44	248	42	211	178	373	44	62	308	894
**87**	8	211	46	298	147	291	74	98	275	898	44	248	42	211	180	376	48	66	314	901
**88**	8	211	46	298	148	292	77	102	279	903	44	248	42	211	182	378	54	72	322	909
**89**	8	211	46	298	148	292	81	106	283	907	44	248	42	211	184	382	59	78	329	919
**90**	8	211	46	298	149	293	84	111	287	913	44	248	42	211	188	386	63	84	337	929
**91**	9	212	46	298	150	294	90	118	295	922	44	248	42	211	191	389	68	88	345	936
**92**	9	212	46	298	150	294	96	123	301	927	44	248	42	211	192	390	70	90	348	939
**93**	9	212	46	298	150	294	102	129	307	933	44	248	42	211	192	390	75	95	353	944
**94**	9	212	46	298	151	295	109	137	315	942	44	248	42	211	195	392	77	97	358	948
**95**	9	212	46	298	152	296	117	147	324	953	44	248	42	211	196	393	79	99	361	951
**96**	9	212	46	298	153	297	124	155	332	962	44	248	42	211	198	395	85	105	369	959
**97**	9	212	46	298	153	297	132	162	340	969	44	248	42	211	199	396	87	107	372	962
**98**	9	212	46	299	154	298	138	168	347	977	44	248	42	211	202	400	88	108	376	967
**99**	9	212	46	299	156	300	144	175	355	986	44	248	42	211	208	406	94	114	388	979
**100**	9	212	46	299	159	303	155	186	369	1000	44	248	43	212	215	413	106	126	408	999

Table S2. Estimation, in a 1000 cases development (A) and external validation (B) cohorts of relative values of missed csPCa and avoided biopsies for different thresholds and PI-RAD categories.

**Series**	DEVELOPMENT COHORT										VALIDATION COHORT									
**Group**	PIRADS 1-2		PIRADS 3		PIRADS 4		PIRADS 5		Global cohort		PIRADS 1-2		PIRADS 3		PIRADS 4		PIRADS 5		Global cohort	
Cases	*csPC*	*total*	*csPC*	*total*	*csPC*	*total*	*csPC*	*total*	*csPC*	*total*	*csPC*	*total*	*csPC*	*total*	*csPC*	*total*	*csPC*	*total*	*csPC*	*total*
	9	*212*	*46*	*299*	*159*	*303*	*155*	*186*	*369*	*1000*	*44*	*248*	*43*	*212*	*215*	*413*	*106*	*126*	*408*	*999*
**Cut-off point**	Missed csPC (%)	Avoided Bx (%)	Missed csPC (%)	Avoided Bx (%)	Missed csPC (%)	Avoided Bx (%)	Missed csPC (%)	Avoided Bx (%)	Missed csPC (%)	Avoided Bx (%)	Missed csPC (%)	Avoided Bx (%)	Missed csPC (%)	Avoided Bx (%)	Missed csPC (%)	Avoided Bx (%)	Missed csPC (%)	Avoided Bx (%)	Missed csPC (%)	Avoided Bx (%)
**0**	0	0	0	0	0	0	0	0	**0**	**0**	0	0	0	0	0	0	0	0	**0**	**0**
**1**	11	22	0	3	0	0	0	0	**0**	**6**	7	18	0	3	0	0	1	1	**1**	**5**
**2**	33	47	0	5	0	0	0	0	**1**	**12**	9	35	0	5	0	0	1	2	**1**	**10**
**3**	33	62	0	7	0	0	0	0	**1**	**15**	23	48	2	8	0	0	1	2	**3**	**14**
**4**	56	71	2	11	0	0	0	0	**2**	**19**	32	61	5	12	0	1	1	2	**4**	**18**
**5**	56	77	2	17	0	0	0	0	**2**	**22**	34	65	7	17	0	2	1	2	**5**	**21**
**6**	56	82	2	21	0	1	0	0	**2**	**24**	41	73	7	21	0	2	1	2	**6**	**23**
**7**	56	86	11	26	0	1	0	0	**3**	**26**	45	77	9	27	0	2	1	2	**6**	**26**
**8**	56	88	15	31	0	1	0	0	**3**	**28**	52	81	9	33	0	3	1	2	**7**	**29**
**9**	56	91	15	35	0	1	0	0	**3**	**30**	59	83	9	37	0	3	1	2	**8**	**30**
**10**	56	92	20	39	1	2	0	0	**4**	**32**	68	87	9	42	0	4	1	2	**9**	**32**
**11**	56	92	24	45	1	3	0	1	**5**	**34**	73	90	12	48	0	4	1	2	**10**	**35**
**12**	56	94	24	51	1	4	0	1	**5**	**36**	75	91	14	52	1	5	1	2	**10**	**36**
**13**	67	95	24	56	1	4	0	1	**5**	**38**	77	91	14	58	1	6	1	2	**11**	**38**
**14**	67	95	28	59	1	4	0	1	**5**	**39**	80	92	14	62	1	6	1	2	**11**	**39**
**15**	67	96	28	62	1	4	0	1	**5**	**40**	82	94	14	63	2	7	1	2	**12**	**40**
**16**	78	97	33	66	1	4	1	1	**7**	**42**	82	94	19	67	2	9	1	2	**12**	**42**
**17**	78	97	35	68	1	5	1	1	**7**	**42**	86	95	23	69	2	10	1	2	**13**	**43**
**18**	78	97	35	70	2	6	1	1	**7**	**43**	89	96	30	71	2	11	1	2	**14**	**44**
**19**	78	98	35	72	2	7	1	1	**7**	**45**	91	97	33	73	2	13	1	2	**14**	**45**
**20**	78	98	43	75	2	8	1	1	**8**	**46**	91	97	33	75	2	13	1	2	**14**	**46**
**21**	78	98	46	77	3	9	1	2	**9**	**47**	91	97	37	77	2	15	1	2	**15**	**47**
**22**	78	98	52	79	3	10	1	2	**10**	**48**	91	97	37	79	3	15	1	2	**15**	**47**
**23**	78	99	59	81	3	10	1	2	**11**	**48**	91	97	42	82	3	17	1	2	**16**	**49**
**24**	78	99	59	81	3	11	1	2	**11**	**49**	91	97	44	83	4	18	1	2	**17**	**50**
**25**	78	99	61	83	3	12	1	2	**11**	**50**	91	98	44	83	4	19	1	2	**17**	**50**
**26**	78	99	67	86	3	13	1	2	**12**	**51**	93	98	44	83	5	21	1	2	**17**	**51**
**27**	78	99	67	87	4	15	1	2	**12**	**52**	93	98	44	85	5	21	1	2	**17**	**51**
**28**	89	99	67	88	4	15	1	2	**12**	**52**	93	98	47	86	6	23	1	2	**18**	**52**
**29**	89	99	70	89	4	17	1	2	**13**	**53**	93	98	49	87	7	24	1	2	**19**	**53**
**30**	89	99	72	90	6	19	1	2	**14**	**54**	93	98	53	89	8	24	1	2	**20**	**54**
**31**	89	100	76	91	6	19	1	2	**14**	**54**	93	98	67	91	8	25	1	2	**22**	**54**
**32**	89	100	78	91	6	20	1	2	**15**	**55**	95	99	72	92	9	26	1	2	**23**	**55**
**33**	89	100	78	91	7	21	1	2	**15**	**55**	95	99	72	94	9	27	1	2	**23**	**56**
**34**	89	100	80	93	8	21	1	2	**16**	**56**	95	99	72	94	10	28	1	3	**23**	**56**
**35**	89	100	80	93	8	22	1	2	**16**	**56**	95	99	74	94	10	29	1	3	**24**	**57**
**36**	89	100	83	93	8	22	1	2	**16**	**56**	95	99	79	95	11	30	1	3	**25**	**58**
**37**	89	100	87	94	8	23	1	2	**17**	**57**	95	99	79	95	11	31	1	3	**25**	**58**
**38**	89	100	89	95	10	24	1	2	**18**	**57**	95	99	79	95	11	31	1	3	**25**	**58**
**39**	89	100	93	95	11	26	1	2	**19**	**58**	95	99	79	95	12	33	1	3	**25**	**59**
**40**	89	100	93	95	12	28	1	2	**19**	**59**	95	99	84	96	12	33	1	4	**26**	**59**
**41**	89	100	93	95	13	30	1	2	**20**	**59**	95	99	88	97	14	35	1	4	**27**	**60**
**42**	89	100	93	95	14	31	1	2	**20**	**59**	95	99	88	98	15	37	1	4	**28**	**61**
**43**	89	100	93	96	14	32	1	2	**20**	**60**	98	100	88	98	16	38	1	4	**28**	**62**
**44**	89	100	93	96	14	33	1	2	**20**	**60**	98	100	88	98	16	39	1	4	**28**	**62**
**45**	89	100	93	96	14	34	1	2	**20**	**61**	98	100	88	98	17	40	1	4	**29**	**62**
**46**	89	100	93	97	16	36	1	2	**21**	**61**	98	100	91	98	20	43	1	4	**31**	**64**
**47**	89	100	93	97	18	38	2	3	**22**	**62**	98	100	91	98	21	44	1	5	**31**	**64**
**48**	89	100	93	97	20	41	2	3	**23**	**63**	98	100	91	98	22	45	2	6	**32**	**65**
**49**	89	100	93	97	21	43	2	3	**24**	**64**	100	100	91	98	23	47	2	6	**33**	**66**

**50**	89	100	93	97	23	45	3	4	**25**	**64**	100	100	91	98	24	49	2	6	**34**	**67**
**51**	89	100	93	97	25	47	3	4	**25**	**65**	100	100	91	98	27	51	2	6	**35**	**67**
**52**	89	100	93	97	27	49	3	4	**27**	**66**	100	100	91	98	29	52	2	6	**36**	**68**
**53**	89	100	96	97	28	50	3	4	**27**	**66**	100	100	91	98	32	54	2	6	**38**	**69**
**54**	89	100	96	97	28	50	3	4	**27**	**66**	100	100	91	98	33	55	3	8	**38**	**69**
**55**	89	100	96	98	30	52	3	4	**28**	**67**	100	100	91	98	33	56	4	10	**39**	**70**
**56**	89	100	96	98	31	54	3	4	**29**	**68**	100	100	91	98	34	57	4	10	**39**	**71**
**57**	89	100	96	98	33	56	3	5	**30**	**68**	100	100	91	98	36	59	5	10	**40**	**71**
**58**	89	100	96	98	35	58	3	5	**30**	**69**	100	100	91	98	37	60	5	10	**41**	**72**
**59**	89	100	96	98	37	59	3	6	**31**	**70**	100	100	91	98	39	62	6	11	**42**	**73**
**60**	89	100	96	99	40	61	3	6	**33**	**70**	100	100	91	98	40	63	6	11	**43**	**73**
**61**	89	100	98	99	42	63	5	8	**34**	**71**	100	100	93	99	41	64	7	13	**44**	**74**
**62**	89	100	98	99	44	64	5	8	**35**	**72**	100	100	95	99	43	66	7	13	**45**	**75**
**63**	89	100	98	99	46	66	6	11	**37**	**73**	100	100	95	99	43	67	9	15	**46**	**75**
**64**	89	100	100	99	48	67	6	11	**38**	**73**	100	100	95	99	45	68	9	15	**47**	**76**
**65**	89	100	100	99	50	69	6	12	**39**	**74**	100	100	95	99	46	68	10	17	**48**	**76**
**66**	89	100	100	99	52	71	6	13	**40**	**75**	100	100	95	99	50	71	10	17	**50**	**77**
**67**	89	100	100	99	57	74	7	14	**42**	**76**	100	100	95	99	50	71	11	17	**50**	**78**
**68**	89	100	100	99	59	76	7	14	**43**	**76**	100	100	95	99	53	73	12	18	**52**	**78**
**69**	89	100	100	99	61	77	8	15	**44**	**77**	100	100	95	99	53	73	13	20	**52**	**79**
**70**	89	100	100	99	63	78	9	16	**46**	**77**	100	100	95	99	54	74	13	20	**53**	**79**
**71**	89	100	100	99	65	79	9	17	**47**	**78**	100	100	95	99	56	76	13	20	**54**	**80**
**72**	89	100	100	99	67	81	10	17	**47**	**78**	100	100	95	99	59	77	14	21	**55**	**80**
**73**	89	100	100	100	68	82	10	17	**48**	**79**	100	100	95	99	60	77	15	23	**56**	**81**
**74**	89	100	100	100	72	83	12	19	**50**	**80**	100	100	95	99	62	79	16	24	**58**	**81**
**75**	89	100	100	100	75	86	13	20	**53**	**81**	100	100	95	99	63	80	16	24	**58**	**82**
**76**	89	100	100	100	77	87	15	22	**54**	**81**	100	100	95	99	65	81	19	27	**60**	**83**
**77**	89	100	100	100	78	88	15	23	**55**	**82**	100	100	95	99	66	81	19	27	**61**	**83**
**78**	89	100	100	100	81	89	18	25	**57**	**83**	100	100	95	99	67	82	22	29	**62**	**83**
**79**	89	100	100	100	83	91	19	26	**59**	**83**	100	100	98	100	70	84	24	31	**64**	**84**
**80**	89	100	100	100	85	92	22	28	**60**	**84**	100	100	98	100	71	85	25	32	**65**	**85**
**81**	89	100	100	100	87	93	26	32	**63**	**85**	100	100	98	100	72	85	25	33	**66**	**85**
**82**	89	100	100	100	88	94	28	33	**64**	**86**	100	100	98	100	75	86	25	33	**67**	**86**
**83**	89	100	100	100	89	94	32	37	**66**	**86**	100	100	98	100	77	88	30	38	**70**	**87**
**84**	89	100	100	100	90	95	35	41	**68**	**87**	100	100	98	100	81	90	31	40	**72**	**88**
**85**	89	100	100	100	91	95	39	45	**70**	**88**	100	100	98	100	82	90	34	42	**73**	**88**
**86**	89	100	100	100	92	96	43	48	**72**	**89**	100	100	98	100	83	90	42	49	**75**	**89**
**87**	89	100	100	100	92	96	48	53	**75**	**90**	100	100	98	100	84	91	45	52	**77**	**90**
**88**	89	100	100	100	93	96	50	55	**76**	**90**	100	100	98	100	85	92	51	57	**79**	**91**
**89**	89	100	100	100	93	96	52	57	**77**	**91**	100	100	98	100	86	92	56	62	**81**	**92**
**90**	89	100	100	100	94	97	54	60	**78**	**91**	100	100	98	100	87	93	59	67	**83**	**93**
**91**	100	100	100	100	94	97	58	63	**80**	**92**	100	100	98	100	89	94	64	70	**85**	**94**
**92**	100	100	100	100	94	97	62	66	**82**	**93**	100	100	98	100	89	94	66	71	**85**	**94**
**93**	100	100	100	100	94	97	66	69	**83**	**93**	100	100	98	100	89	94	71	75	**87**	**94**
**94**	100	100	100	100	95	97	70	74	**85**	**94**	100	100	98	100	91	95	73	77	**88**	**95**
**95**	100	100	100	100	96	98	75	79	**88**	**95**	100	100	98	100	91	95	75	79	**88**	**95**
**96**	100	100	100	100	96	98	80	83	**90**	**96**	100	100	98	100	92	96	80	83	**90**	**96**
**97**	100	100	100	100	96	98	85	87	**92**	**97**	100	100	98	100	93	96	82	85	**91**	**96**
**98**	100	100	100	100	97	98	89	90	**94**	**98**	100	100	98	100	94	97	83	86	**92**	**97**
**99**	100	100	100	100	98	99	93	94	**96**	**99**	100	100	98	100	97	98	89	90	**95**	**98**
**100**	100	100	100	100	100	100	100	100	**100**	**100**	100	100	100	100	100	100	100	100	**100**	**100**

(**A**)

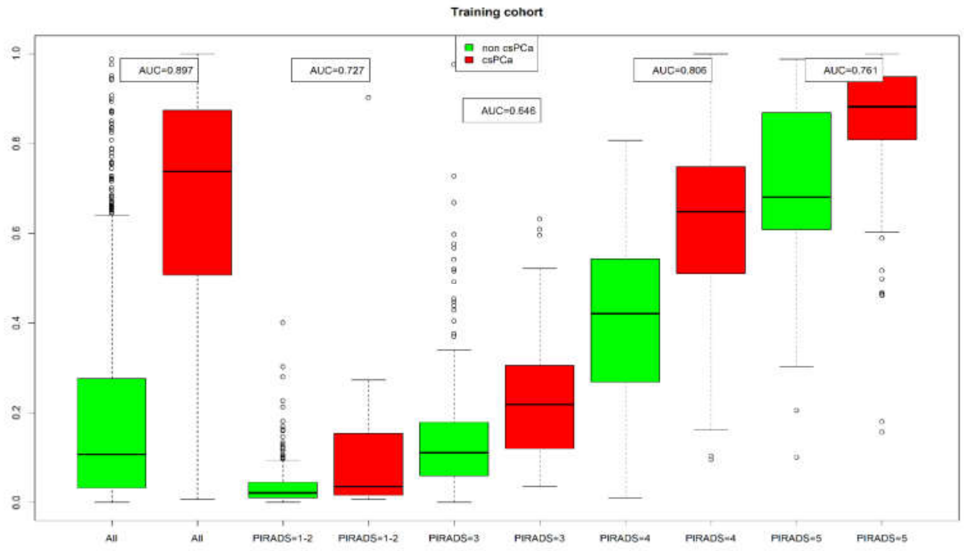

(**B**)

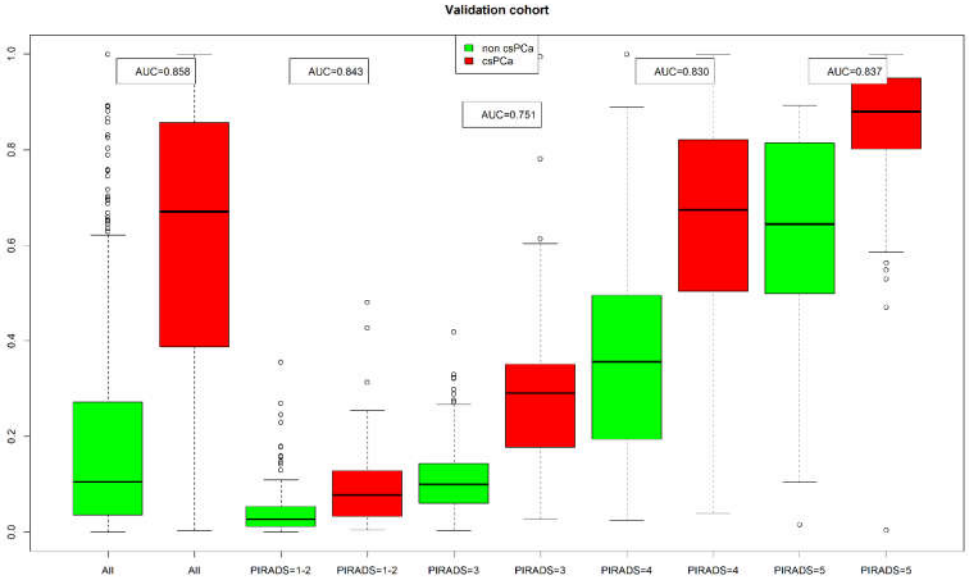

Figure S3. Boxplots of the likelihoods of csPCa in overall men and regarding PI-RADS categories in development cohort (**A**) and external validation cohort (**B**).

(**A**)

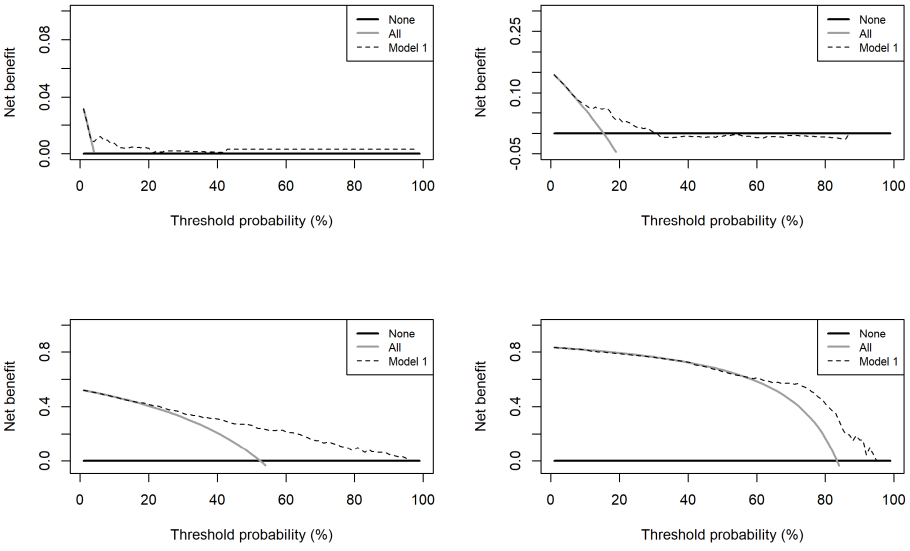

(**B**)

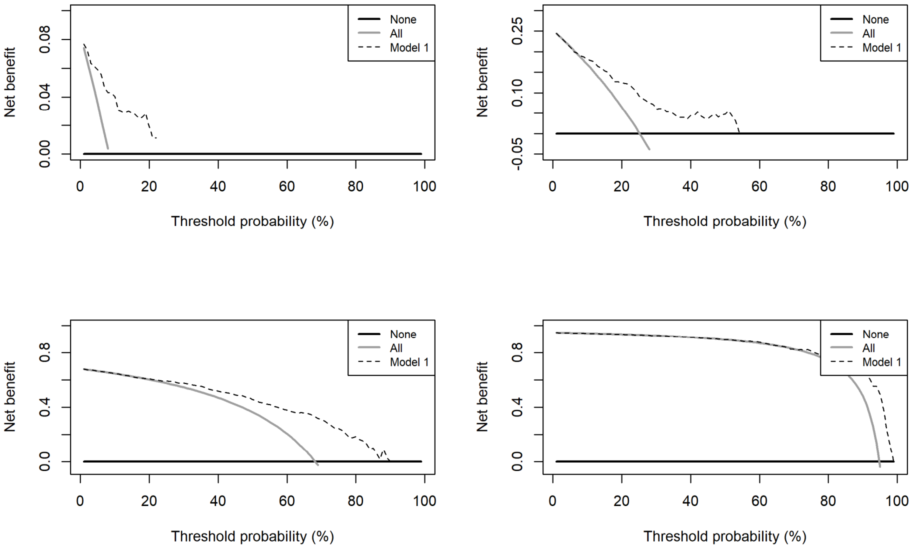

Figure S4. DCA showing the net benefit of MRI-PM biopsying all men according to the PI-RADS categories <3 (upper left), 3 (upper right), 4 (lower left), and 5 (lower right) in development cohort (**A**) and external validation cohort (**B**).
